# Impact of surgical checklists on the time of surgical processes: a cross-sectional study

**DOI:** 10.1590/0100-6991e-20233425-en

**Published:** 2023-01-24

**Authors:** JOSEMAR BATISTA, ELAINE DREHMER DE ALMEIDA CRUZ, DANIELI PARREIRA DA SILVA, SAIMON DA SILVA NAZÁRIO, BÁRBARA CRIS SKORA ANTUNES

**Affiliations:** 1 - Universidade Federal do Paraná, Programa de Pós-graduação em Enfermagem - Curitiba - PR - Brasil

**Keywords:** Checklist, Medical Errors, Quality Indicators, Health Care, Surgery Department, Hospital, Patient Safety, Lista de Checagem, Erros Médicos, Indicadores de Qualidade em Assistência à Saúde, Centro Cirúrgico Hospitalar, Segurança do Paciente

## Abstract

**Objectives:**

to analyze the impact of the use of checklists on the mean time of the operative processes of patients undergoing hip and knee arthroplasties.

**Method:**

cross-sectional and analytical research conducted between November/2020 and March/2022 with retrospective consultation in a simple random sample of 291 medical records, distributed in three periods (2010/2013/2016). Descriptive and inferential statistics were used for data analysis; p=0.05 values indicated significance.

**Results:**

there was a reduction in the time of entry-exit from the operating room (p=0.002), surgery (p<0.001) and between the onset-anesthesia and the beginning-incision (p=0.021). There was no difference in time between patients with and without the use of checklists (p=0.05) in relation to the variables onset-anesthesia, onset-incision, time of anesthesia and surgery.

**Conclusion:**

the implementation of checklists potentially contributed to reduce the time of use of the operating room. The nonassociation of its use with the increase in the mean time of the processes in the operating room shows that its application does not interfere negatively in this indicator.

## RESUMO

**Objetivo:**

analisar o impacto do uso de checklists no tempo médio dos processos operatórios de pacientes submetidos a artroplastias de quadril e joelho.

**Método:**

pesquisa transversal e analítica realizada entre novembro/2020 e março/2022 com consulta retrospectiva em amostra aleatória simples de 291 prontuários, distribuídos em três períodos (2010/2013/2016). Utilizou-se estatística descritiva e inferencial para análise dos dados; valores de p=0,05 indicaram significância.

**Resultados:**

houve redução do tempo de entrada-saída da sala cirúrgica (p=0,002), de cirurgia (p<0,001) e entre o início-anestesia e início-incisão (p=0,021). Não houve diferença no tempo entre os pacientes com e sem o uso dos checklists (p=0,05) com relação às variáveis início-anestesia, início-incisão, tempo de anestesia e de cirurgia.

**Conclusão:**

a implantação de checklists potencialmente contribuiu para reduzir o tempo de uso da sala cirúrgica. A não associação de seu uso ao aumento do tempo médio dos processos no centro cirúrgico mostra que sua aplicação não interfere negativamente nesse indicador.

## INTRODUCTION

While technological advances in healthcare allow to incorporate new surgical approaches into professional practice^1^, there is a marked care risk and interest of managers, researchers, and health professionals in implementing actions to offer safe and good surgical and anesthetic care^2^. Considering that the quality of care is an important component of the performance of the health system, effectiveness, accessibility, and equity are essential to provide timely, efficient, safe, and people-centered care, with a view towards improving outcomes^3^ ^-^ ^4^.

In the surgical specialty, although there have been significant advances, there is evidence that mortality is higher among patients who receive low-quality care than due to lack of access to services^5^. The Surgical Safety Checklist, in checklist format, indicated by the World Health Organization and recommended by the Safe Surgery Saves Lives Program, is one of the tools used to minimize the problems that lead to incidents in the professional-system interaction and provide improvements in the process of the surgical team, achieving the goals established by quality management and strengthening the excellence of care^6^ ^-^ ^7^. Its use has proven to be an acceptable cost-effective strategy to promote patient safety, the surgical process, and results^8^ ^-^ ^9^.

In the hospital of this research, in the biennium 2010-2011, the checklist proposed by the WHO was adapted for use in the operating room, the specialty orthopedics being the pioneer in its application^10^. In 2014, another checklist was implemented for use in inpatient units, applicable in the pre and postoperative phases, whose objectives were to verify safety items before the patient is transferred to the operating room^11^. However, conducting research to investigate the impact of interventions on patient safety does not seem to have accompanied the speed of implementation of different tools, in favor of improvements in care indicators and surgical results, with emphasis on orthopedics, known to be of higher risk, probability of errors, and deaths^12^.

The growing need to optimize operating room processes and measure the time an operating room is used are among the institutions’ efforts to improve system efficiency^13^. Through the history of the development and implementation of surgical checklists in the study hospital, and the relevance of analyzing the results of processes to promote quality of care, the objective of this research was to analyze the impact of the use of checklists on the average time of the operative processes of patients undergoing hip and knee arthroplasties

## METHOD

This is a cross-sectional and analytical research, with a quantitative approach, carried out in a large teaching hospital in the southern region of Brazil. Data collection took place between November 2020 and March 2022, and considered data from hospitalizations in different time intervals and corresponding to the periods before and after implementation of two types of surgical safety checklist.

In 2011, a checklist applicable to the operating room was implemented, consisting of 45 items, organized into four moments: patient reception, before anesthetic induction, before the surgical incision, and before the patient leaves the operating room^10^. In 2014, a checklist was developed and implemented the preoperative and immediate and mediate postoperative periods, applicable to surgical hospitalization units, with 97 safety items^11^.

Data from this research were extracted from the thesis under construction, whose objective is to evaluate the effects of implementing checklists in the reduction of adverse events in patients undergoing hip and knee arthroplasties in a Brazilian teaching hospital. For the sample calculation, the result of a pilot test was considered to estimate the prevalence of adverse events in the pre- and post-intervention periods. The significance level of 5% and test power of 80% resulted in a minimum sample size of 97 medical records for each checklist implementation period (2010/2013/2016). Simple random selection of eligible medical records was performed from the hospital’s database; cases unavailable in the medical archive service were replaced by the immediately subsequent medical records of the general list of arthroplasties performed in the period from January 1^st^ to December 31^st^ of each year of the study.

Inclusion criteria were patients aged ≥18 years and with a minimum hospital stay of 24 hours. Cases of intraoperative deaths and length of stay <24 hours were included. Cases with diagnoses related to psychiatric illnesses, as previously established, were excluded^14^ ^-^ ^15^. Data collection was carried out by a primary reviewer, with a retrospective consultation of medical records and analysis of the records contained in the pre-anesthetic records, anesthetic and surgical descriptions, and the systematization of perioperative nursing care, referring to the first surgical procedure and corresponding to the analyzed hospitalization (index admission).

These documents were used as a basis for extracting information and filling out a semi-structured form, designed for this research, in order to characterize the demographic, clinical, surgical, and anesthetic profile, and obtain: (1) the patient’s length of stay in the operating room (hours); (2) time of anesthetic duration (hours); (3) time of surgical duration (hours); and (4) time between onset of anesthesia and surgical incision (minutes).

In order to quantify the completeness of the checklist items, the documents were classified as: (A) absence/non-completion of the checklist; (B) complete checklist; and (C) partial completion of the checklist.

The collected data were entered, by double typing, in a Microsoft Office Excel 2016^®^ spreadsheet and, after checking for inconsistencies, they were analyzed with statistical assistance and use of IBM SPSS 20 software (Statistical Package for the Social Sciences).

Quantitative variables were described by univariate descriptive statistics and categorical variables were described by absolute and relative frequencies. To compare the quantitative variables between the pre-intervention, intervention I and intervention II (2010/2013/2016) periods, we used the one-way analysis of variance (ANOVA) model or the non-parametric Kruskal-Wallis test. Multiple comparisons were performed using the Dunn’s post-hoc test, with Bonferroni-corrected p-values. To compare the duration of the operative processes between patients, with and without a checklist, we applied the non-parametric Mann-Whitney test. We evaluated the normality of the quantitative variables with Kolmogorov-Smirnov test. Values of p≤0.05 indicated statistical significance.

The research was approved by the institutional ethics committee under opinion number 3,651,686.

## RESULTS

We evaluated 291 medical records of patients undergoing hip and/or knee arthroplasties, with a mean age of 57.2 years (±14.4), 56.5 (±15.5), and 59.5 (±15.4), in 2010, 2013, and 2016, respectively. Table 1 shows patients’ demographic, clinical, and surgical-anesthetic profile. 

**Table 1:** Distribution of the demographic, clinical, surgical, and anesthetic profile of patients undergoing hip and knee arthroplasties. Curitiba, PR, 2022.

Variable	Year		
	2010 (n=97)	2013 (n=97)	2016 (n=97)
	n (%)	n (%)	n (%)
Sex			
Female	56 (57.7)	59 (60.8)	62 (63.9)
Male	41 (42.3)	38 (39.2)	35 (36.1)
Comorbidities /Risk factors*			
Systemic arterial hypertension	58 (59.8)	50 (51.6)	56 (57.7)
Smoking	15 (15.5)	25 (25.8)	13 (13.4)
Diabetes mellitus	12 (12.4)	14 (14.4)	19 (19.6)
Lung disease^†^	10 (10.3)	6 (6.2)	6 (6.2)
Hypothyroidism/Hyperthyroidism	9 (9.3)	11 (11.3)	7 (7.2)
Rheumatoid arthritis	6 (6.2)	3 (3.1)	3 (3.1)
Heart disease	6 (6.2)	7 (7.2)	7 (7.2)
Hepatitis	5 (5.2)	7 (7.2)	4 (4.1)
Dyslipidemia	4 (4.1)	2 (2.1)	5 (5.2)
Alcoholism	4 (4.1)	4 (4.1)	6 (6.2)
Hemophilia	3 (3.1)	10 (10.3)	8 (8.3)
Other^‡^	4 (4.2)	4 (4.2)	1 (1.0)
Preoperative diagnosis			
Gonarthrosis	36 (37.1)	37 (38.1)	46 (47.4)
Coxarthrosis	32 (33)	34 (35.1)	36 (37.1)
Aseptic component loosening	12 (12.4)	8 (8.3)	8 (8.3)
Secondary coxarthrosis	7 (7.2)	3 (3.1)	2 (2.1)
Secondary gonarthrosis	4 (4.1)	11 (11.3)	3 (3.1)
Other^§^	6 (6.2)	4 (4.1)	2 (2)
Surgery			
Total knee arthroplasty	37 (38.1)	45 (46.4)	51 (52.6)
Total hip arthroplasty	44 (45.4)	43 (44.3)	36 (37.1)
Hip revision arthroplasty	9 (9.3)	8 (8.3)	6 (6.2)
Knee revision arthroplasty	7 (7.2)	1 (1)	4 (4.1)
Variable	Year		
	2010 (n=97)	2013 (n=97)	2016 (n=97)
	n (%)	n (%)	n (%)
Preoperative length of stay			
<24	91 (93.8)	88 (90.7)	93 (95.9)
>24	6 (6.2)	9 (9.3)	4 (4.1)
Surgical classification			
Elective	95 (97.9)	97 (100)	97 (100)
Emergency	2 (2.1)	0 (0)	0 (0)
Degree of contamination			
Clean	97 (100)	97 (100)	96 (99)
Infected	0 (0)	0 (0)	1 (1)
ASA Surgical Risk^||^			
I	22 (22.7)	14 (14.4)	12 (12.4)
II	61 (62.9)	72 (74.2)	70 (72.2)
III	14 (14.4)	11 (11.3)	15 (15.5)
Type of anesthesia^¶^			
Epidural	86 (88.7)	65 (67)	7 (7.2)
Spinal	73 (75.3)	74 (76.3)	70 (72.2)
Sedation	71 (73.2)	61 (62.9)	50 (51.6)
General	21 (21.7)	23 (23.7)	43 (44.3)
Location/Block	0 (0)	3 (3.1)	4 (4.1)

*The same patient could have more than one risk factor/comorbidity; †asthma, bronchitis, acute pulmonary edema, pulmonary emphysema, and chronic obstructive pulmonary disease; ‡osteoporosis and neoplasms; §femoral neck fracture, fracture of synthesis material, femoral osteonecrosis, periprosthetic fracture, femoral fracture, hemophilic arthropathy, and operative infection; ^||^American Society of Anesthesiology; ^¶^The same patient can undergo more than one type of anesthesia.

Table 2 shows the duration of the surgery-related processes, before and after the implementation of the surgical checklists.

**Table 2:** Distribution of the time analysis of the surgery-related processes, according to the period of implementation of the checklists. Curitiba, PR, 2022.

Variable	Period	n	Average	Standard deviation	Median	Minimum	Maximum	p-value*
Time of operating room (hours)	2010	97	3.4	1.0	3.3	1.3	6.8	0.002
	2013	97	3.4	1.4	3.1	0.5	10.5	
	2016	97	3.0	0.7	2.9	0.9	4.9	
Time of anesthesia (hours)	2010	97	3.1	1.0	2.9	1.3	6.5	0.078
	2013	97	3.3	1.4	2.9	0.5	10.4	
	2016	97	2.8	0.7	2.8	0.6	4.7	
Variable	Period	n	Average	Standard deviation	Median	Minimum	Maximum	p-value*
Surgical time (hours)	2010	97	2.3	0.9	2.2	0.7	5.5	<0.001
	2013	97	2.2	1.2	2.0	0.3	8.3	
	2016	97	1.8	0.6	1.8	0.3	3.9	
Time from start of anesthesia to start of surgery (minutes)	2010	97	49.7	18.3	50	0	100	0.022
	2013	97	48.9	20.6	47	0	125	
	2016	97	44.6	15.6	45	15	100	

*Kruskal-Wallis non-parametric test, p<0.05.

Among the variables that showed a significant difference, there was a reduction in the time to enter and leave the operating room, between the beginning of anesthesia and surgery, and in the operative time between 2010 and 2016 (p<0.05), as shown in Table 3. There was no difference in time in the other comparisons.

**Table 3:** Distribution of the p-value according to the time of the surgical process and the periods of comparison referring to the implementation of checklists. Curitiba, PR, 2022.

Periods compared	p-value*		
	Room entry - exit time (hours)	Surgery time (hours)	Time from start of anesthesia to start of surgery (minutes)
2010 x 2013	0,332	0,247	1
2010 x 2016	0,002	<0,001	0,021
2013 x 2016	0,182	0,127	0,209

*Dunn ‘s test, p<0.05 (p-values corrected by Bonferroni).

Regarding the completeness of all verified documents, in 2013 89.7% (n=87) of the checklists were absent/not completed, 9.3% (n=9) were partially filled, and 1% (n=1) was complete. In 2016, there was no checklist in 43.3% (n=42), partial completion in 45.4% (n=44), and completion in 11.3% (n=11). For the checklist applied in the inpatient units, 5.2% (n=5) of the instruments were absent and 94.9% (n=92) were partially completed.

There was no significant difference between the duration of anesthesia and the duration of surgery between patients with and without the use of checklists (p≥0.05) (Table 4).

**Table 4:** Distribution of anesthesia and surgical time of admissions with and without the use of checklists. Curitiba, PR, 2022..

Variable	Use of Checklist	n	Average	Standard deviation	Median	Minimum	Maximum	p-value*
Time with checklist 1								
Time between onset and end of anesthesia (hours)	No Yes	87 10	3.3 3.1	1.4 0.9	2.9 2.9	0.5 2.0	0.5 2.0	0.995
Surgical time (hours)	No Yes	87 10	2.2 2.1	1.2 0.7	2.0 1.9	0.3 1.3	8.3 3.4	0.921
Time from start of anesthesia to start of surgery (minutes)	No Yes	87 10	48.7 51.0	21.3 13.1	45.0 55.0	0.0 20.0	125.0 65.0	0.310
Time with checklist 1 and 2								
Time between onset and end of anesthesia (hours)	None or only one Both	46 51	2.7 2.9	0.7 0.8	2.8 2.8	0.6 0.8	4.0 4.7	0.558
Surgical time (hours)	None or only one Both	46 51	1.7 1.9	0.6 0.7	1.8 1.8	0.3 0.3	3.0 3.9	0.136
Time from start of anesthesia to start of surgery (minutes)	None or only one Both	46 51	45.6 43.8	13.2 17.6	45.0 40.0	20.0 15.0	70.0 100.0	0.298

*Mann-Whitney non-parametric test, p<0.05.

## DISCUSSION

The results of this analysis show improvements in the “time” indicator with the use of checklists applied in the operating room and inpatient units. This finding is in line with efforts to maximize efficiency in the use of the operating room and its secondary effects, such as improvements in patient safety, productivity in the sector, satisfaction of the health team, patients and families, and reduction of hospital costs^16^.

When observing the results of the surgical process before and after the implementation of the checklists, there was a reduction (p<0.05) in the average time the patient spent in the operating room, between the beginning of anesthesia and the beginning of surgery, and of surgical time. This data differs from that found in a North American study with an analysis of 7,265 orthopedic surgeries, in which no significant difference was observed in the length of stay of the patient in the operating room and in operative time^17^. A different result was also reported in an Australian investigation about the impacts of introducing a checklist, which did not identify differences in the length of stay in the operating room until the beginning of the orthopedic procedure, nor in the time between the end of the surgery and the patient’s departure from the room^18^.

The positive results of this research regarding the shorter duration of the surgical processes possibly reflect the consolidation of the safe surgery program at the teaching hospital, which began more than a decade ago. In addition, they are consistent with the strengthening of dimensions of the patient safety culture verified among professionals working in the surgical units and operating room at the same institution and, in particular, those related to communication and adequacy of human resources^19^.

Checklists improve communication between teams and provide opportunities for dialogue, with the sharing of relevant information for safety and quality of care^20^. These benefits contribute to the planning of the perioperative care steps, especially in the period prior to surgery, with the verification of items necessary for the anesthetic-surgical act. Those indispensable for the beginning of the procedure stand out, such as test results and surgical authorization documents, as well as the availability of materials and equipment necessary for the safe course of the procedure, avoiding delays resulting from handling and preoperative management problems, reflecting in the shorter length of stay of the patient in the operating room and in the hospital.

The good performance of the operating room management processes is reflected in the care quality indicators and guarantees the organization’s sustainability^21^. The impact of checklists on patient safety must be preceded by improvements in work processes in the operating room^22^, as shown in this research, a potential result of the safe surgery program, including the implementation of verification instruments.

In this context, patient safety policies or programs cooperate in the rise of quality indicators, as they improve the performance of the surgical, nursing, and anesthesiology teams. The Genesis Health System recommends the expansion of these programs, based on the principles of highly reliable organizations, as a central institutional value and with the aim of obtaining good care results, with a reduction in deaths and adverse events^23^.

A study shows that the routine use of checklists contributes to the familiarization of the surgical team in the execution of the steps, optimizing surgical care and reducing operative and hospitalization time^24^. In this research, the average surgery time increased from 138 minutes to 108 minutes. However, it is still superior to the analysis of 165,474 hip or knee arthroplasties, and whose results showed an operative time of approximately 92 minutes^25^. The same study showed that an increase in operative time by 15 minutes increased the risk of surgical site infections, sepsis, and renal failure, as well as hospital readmission and prolonged hospital stay (≥4 days)^25^. Another investigation, conducted with 11,840 knee arthroplasties, showed that surgical time greater than 121 minutes was a predictive factor for surgical wound infections^26^.

We found no significant differences between the duration of anesthesia and the duration of surgery between patients with and without the use of checklists. In 2013, the time from beginning of anesthesia to beginning of surgery was higher among patients who used the checklist, which may be related to the greater severity and complexity of patients, but also to the time spent to carry out the checking steps before induction anesthetic (Sign in) and surgical incision (Time Out).

It is estimated that applying the checklist can be completed in three minutes^27^. Nonetheless, this time may have been longer in the study hospital, as the application depends on the assimilation of the tool, which was recently implemented during the analysis period. The opposite occurred in 2016, when there was a shorter time from beginning of anesthesia to beginning of surgery, and longer anesthetic-surgical time among patients with completion (partial or total) of the checklists. Researchers from Italy identified an association between length of hospital stay and adherence to the checklist, mainly justified by the clinical conditions of patients that result in greater clinical risk and, successively, in the occurrence of adverse events and prolonged hospitalization time^28^.

We highlight the low completeness of the checklist applied in the operating room in 2013, and little improvement in partial or total completion for the year 2016, including for the instrument applied in the inpatient units. This result reiterates that the implementation of surgical safety checklists takes time and requires persistence and strategic planning^22^. Adherence to the application of the checklist and its completeness are related to the value attributed by the team and to the institution’s management processes. The maturity of the patient safety culture and management requirements potentially contribute to routine use and, consequently, to the promotion of quality of care.

When considering that the checklists in the hospital under study were designed, for the most part, by manager nurses and had little involvement of other professional categories, especially surgeons and anesthesiologists, this circumstance is inferred as a possible limiting factor for routine use. We believe that involving the entire interdisciplinary team to design tools that aim to promote safe practices, such as checklists, is a facilitator for their incorporation. Lack of support from managers and hospital management, lack of training/education, and abruptly implemented checklists are among the barriers to successful implementation^20^, which may explain the low adherence to the instruments. On the other hand, we recognize the barriers imposed to innovations, which are often perceived as fads or additional tasks.

The low completeness of surgical checklists during the implantation periods is the main limitation of this research. Retrospective analysis of data, which depends on the quality of records, adds to the limitations of studies documentary basis. Despite these, this research helps to highlight the importance of using a checklist as a tool to promote surgical processes, although other factors not restricted to the analyzed variables may have contributed to improvements in the time indicator.

Routine audits and training/qualification regarding the use and completeness of checklists and the potential results for surgical practice, with emphasis on identifying weaknesses and actions that promote process efficiency, are fundamental in the pursuit of excellence in perioperative care.

## CONCLUSION

The implementation of the checklists potentially had an impact on the reduction of the operating room entrance-exit time, surgery, and between anesthesia start and incision. We identified no association between the use of checklists and the increase in the operating room procedures average time.
